# Universal health coverage for refugees and migrants in the twenty-first century

**DOI:** 10.1186/s12916-018-1208-2

**Published:** 2018-11-26

**Authors:** Ibrahim Abubakar, Alimuddin Zumla

**Affiliations:** 1 UCL Institute for Global Health, University College London, London, UK; 2 Division of Infection and Immunity, Centre for Clinical Microbiology, University College London, London, UK; 3 NIHR Biomedical Research Centre, University College London Hospitals NHS Foundation Trust, London, UK

**Keywords:** Refugees, Migrants, Universal health care, Access

## Abstract

Migration is a determinant of health. Tackling the health needs of migrants and refugees will require action at the local, national, and global levels. Over the past 12 months, *BMC Medicine* has published a collection of articles under the title Migrant and Refugee Health (https://www.biomedcentral.com/collections/migrant-and-refugee-health) addressing a range of health issues affecting refugees and migrants in their countries of origin, on transit, and in their destination countries. In light of these articles, we herein discuss the complex and wide-ranging healthcare needs of different refugee groups in their destination countries as well as the need for accessible and culturally appropriate health services.

## Global policy context

Global migration has become a central feature and key determinant of health in the twenty-first century [1]. The United Nation’s Migration Agency reports that, in 2017, there were an estimated 244 million international migrants globally, including 22 million refugees [2]. Since the movement of populations inevitably leads to consequences for healthcare delivery, national governments and international bodies have a responsibility to ensure that, in keeping with their pledged obligations to the Sustainable Development Goals, no migrant or refugee is “left behind.” Additionally, the United Nations has led the development of the Global Compacts on Migration and Refugees, two landmark agreements that ensure further commitment by international bodies and nations to provide high-quality preventive and curative healthcare to these population groups.

Over the last year, *BMC Medicine* has published a collection of articles under the title Migrant and Refugee Health (https://www.biomedcentral.com/collections/migrant-and-refugee-health) addressing the myriad of health and other issues affecting refugees and migrants in their countries of origin, on transit, and in their destination countries.

## Refugee health

These articles highlight the complex and wide-ranging healthcare needs of various refugee groups in their destination countries and show that refugees require accessible and culturally appropriate health services. Factors that determine the varying needs are influenced by where the refugee population originates from, the reason for their forced departure, the nature and added risks of their journeys, the cultural context they encounter on arrival, and the type of health system in their destination country.

In a study in Durban, South Africa, Meyer-Weitz et al. [3] explored the health access perception of predominantly female caregivers of Congolese refugee children and concluded that long waiting times, negative attitudes, and discrimination are key sources of dissatisfaction with primary healthcare. By contrast, in Australia, an analysis of longitudinal data to investigate levels of post migration psychological adjustment among refugee children concluded that the majority of resettled children and adolescents was well adjusted, especially those with better physical health and school attendance, although young refugees reported greater peer difficulties [4]. Another Australian study investigated psychiatric symptoms among resettled refugee women [5], reporting a high risk of mental health disorders in this group, with particular effects observed in those from Sudan or Burma and those with a reported history of more past trauma events and post migration living difficulties. Similarly, a German questionnaire-based study among female refugees from Afghanistan, Syria, Iran, Iraq, Somalia, and Eritrea concluded that gender-specific trauma, including by family members, determines physical and mental health, quality of life, and the ability to integrate post migration [6]. Further, Kizilhan et al. [7] describe the devastating impact of the Iraqi conflict on Yazidis, a religious minority that has borne a disproportionate burden of violence and mental and physical harm, highlighting the urgent need for an integrated medical response. Additionally, a review of systematic reviews on perinatal outcomes among refugee and asylum-seeking women found adverse pregnancy outcomes complicated by poor access and discrimination [8], and identified the need to overcome access barriers and tackle inequalities in this vulnerable population. An infectious disease screening data analysis of the refugees in the UK resettlement program found variations in the levels of disease by country of origin and pre-migration circumstance, concluding that a tailored and targeted program using a risk-based approach may be appropriate [9]. These studies add to our knowledge of the levels of physical and mental ill health in refugee populations, yet more research is needed on interventions for their reduction and to improve prevention.

Within refugee camps, the provision of healthcare is often challenged due to the inadequacy of resources, ongoing security risks, and the diverse health needs of the populations as determined by previous physical and mental trauma. Through assessments in Greek refugee camps, Rojek et al. [10] surmised that useful clinical data are available via consultations and that these data need to be collected and fed into planning to inform outbreak investigation and control. Similarly, another Greek mixed methods study identified high levels of anxiety disorder and prior violence among a largely Syrian refugee population [11].

Internally displaced persons often suffer equally adverse consequences without the protection and rights sometimes afforded to refugees. In Syria, in the context of the challenges of the ongoing conflict, Aburas et al. [12] describe a case study based on a health center providing maternal and child healthcare in collaboration with the Syrian Expatriate Medical Association, illustrating the potential impact of local groups to complement wider international efforts. Additionally, tackling mental health issues in refugees requires both psychological and pharmacological measures; Ostuzzi et al. [13] describe the UNHCR guidelines on pharmacological interventions for non-affective psychosis which, while limited by the quality of underlying evidence, provide robust practical guidance.

## Climate change refugees

A major future driver of global human displacement will be climate change unless urgent action is taken to mitigate its impact. World Bank estimates suggest that up to 140 million people in densely populated regions of the world will be internally displaced by 2050 [14]. Schwertdle et al. [15] analyze a series of case studies on a planned relocation in Papua New Guinea, an immobile or trapped tribal community in Alaska, a forcibly displaced group in South Sudan, rural urban migration in Northern Australia, and temporary and circular mobility in Spain and Colombia to illustrate the range of potential health risks and opportunities in each context. These case studies illustrate strategies to concurrently make health systems more migrant inclusive and resilient to climate change.

## Returning refugees

Where refugees manage to return to their home countries, they are often faced by weak health systems unable to cope with their health needs. Higgins-Steele et al. [16] describe the problems faced by the Afghani health system in coping with the large influx of returnees, highlighting the urgent need for action and support at every tier.

## Other migrant groups

Challenges in the provision of healthcare to other migrant groups are also determined by factors such as whether they are forced or voluntary migrants, the circumstances surrounding their journey, contextual factors, and access issues in the hosting country. Using a respondent-driven questionnaire-based survey, Hamdiui et al. [17] assessed the factors associated with the uptake of hepatitis B screening, identifying opportunities to improve screening through better information provision and education of migrants on hepatitis B infection and the purpose of screening, as well as the need to work with communities to tackle stigma. In another infectious disease study, Al Zahrani et al. [18] highlighted the importance of migration, conflict, and poverty in cross-border transmission of malaria between Saudi Arabia and Yemen.

## Health financing

Ultimately, access to preventive and curative healthcare is the central requirement to “leaving no one behind” in the quest for true health coverage. Nevertheless, migrants and refugees are unfortunately excluded from most national health systems designed to address the needs of citizens. With this in mind, Spiegel et al. [19] explore innovative financing approaches to ensure health coverage for refugees, suggesting solutions such as a Refugee Health Financing Model during the acute phase and health insurance schemes with multi-party input for protracted situations.

## Conclusions

As nation states sign the two Global Compacts on Migration and Refugees, it is ever more critical to continue to develop the evidence base to inform migrant-friendly effective health systems that will ensure universal health coverage. Ultimately, a test for whether the United Nations Sustainable Development Goals have been achieved will be whether the needs of the most vulnerable in society have been addressed. We therefore call on all stakeholders, civil society, and policymakers to hold leaders to account in achieving these goals.
